# Relations of Vaccination, &c., to Small Pox

**Published:** 1853-05

**Authors:** 


*Relations of Vaccination and Innoculation to Small-Pox.*—From a very interesting and valuable paper upon this subject, communicated to the Epidemiological Society of London, by Dr. Waller Lewis, F. G. S., we take the following extracts. The author commenced by considering the various questions in regard to this disease, of which an elucidation is now anxiously called for. He then stated numerous cases illustrative of the protective power of vaccination, and of the superiority of vaccination to inoculation. He then referred to the probability of the fact that measles are rendered milder by vaccination.

"Joler has described an epidemic of measles that took place in Retzat Circle, in Bavaria, in the district where he himself resided. He says that the disease was much milder among the vaccinated than among the unvaccinated; 15 in 52 died among the non-vaccinated, while barely 1 in 300 died among the vaccinated, showing that measles was 86 times more fatal among the former than the latter."

Examples of imperfect vaccination were then dwelt upon, and the author expressed his opinion clearly and decidedly that where well marked cicatrices were not left, the operation should be accounted a failure, and should be repeated. although he owned that this was not the opinion held by many German and English physicians.

Vaccinating from re-vaccinated persons, from those who had been inoculated, and from such as had previously had smal-pox was strongly denounced, as vaccinia must be extremely modified in such cases. The author added:

"When we interest ourselves strongly in the propagation of vaccination, we must guard ourselves from furnishing arms to its adversaries. And is it not furnishing them with arms to employ a virus of which we are not certain?"

A most interesting collection of cases was then read, in which small-pox had attacked the same individual three or four times; among others, the following, that had come under the author's own attention, was narrated:

"Robert, D. a tradesman, living in North Audley st., had small-pox the first time at the time of his birth, his mother suffering from it at her confinement. He was attacked with the disease a second time when a boy at school, between nine and ten years of age. When 18 years of age, he took it, for the third time, from his sister, who died of it. All the attacks were severe, but the last the most so. He lost his hair and his nails, and the skin of his feet; he was blind for several days, and his life was despaired of. However, he is still alive, and not much disfigured. He was never vaccinated nor inoculated. I believe, if again exposed to the disease, he will take it again."

Cases were then adduced to show that several members of the same family appear sometimes to show great susceptibility to take the disease. The following curious case of small-pox in the lower animals was then adduced, the author adding, that any similar well attested cases would be very valuable additions to the facts collected by the Society on this subject:

"The following case was related to me by a lady of rank, on whose veracity I can place the greatest reliance. Some years ago, just after her confinement, she was seized with small-pox. It became necessary to have her breasts drawn, and, as no child could be obtained, recourse was had to a puppy, which answered the purpose. At the usual time the puppy sickened, and had the disease known by the name of the "distemper." It is said that vaccination, when successfully performed on puppies, will almost to a certainty prove a prophylactic against distemper."

Then followed some interesting cases of individuals who could be neither vaccinated nor inoculated. The last cases adduced were of individuals who appeared to have perfect immunity from small-pox.

"I have detailed the case of Robert D., who evidently possesses a strong innate susceptibility to the action o' the small-pox virus, as shown in his having already taken the disease three several times. I have now to draw your attention to a case the most directly opposite to this. Strangely enough, it is that of his own brother, Thomas. From the elder brother Robert, as well as a sister, having taken the small pox, the parents believed that all their children must take the disease, and refused to have the subject of this case vaccinated or inoculated. He was accordingly exposed when a child to the contagion, lying in the same room with his sister, while she was suffering from the disease, as well as waiting on his brother in his second and third attacks. Although since that time he has been several times exposed to the contagion, he has never felt the slightest ill effects from it. * * * Examples of persons possessing a natural immunity from the disease are rather numerous. Dr. Jackson of Philadelphia, saw a man at the small-pox hospital, engaged in laying out and burying the dead, who had never had an attack of the disease. He had been frequently inoculated and vaccinated, but always unsuccessfully. Van Swieten speaks of a physician, 70 years of age, who had practised through numerous epidemics of the disease, but had never taken it. Diemerbrock states that immunity from small-pox was a privilege of his family. It was possessed, he asserts, by his grandfather, grandmother, his father, and himself."

The author drew the following deductions from the cases adduced:

"1. That vaccination is a most eminent protection against small-pox.

"2. That when perfectly performed it is almost, and, in some instances, more protective, than inoculation or small-pox itself.

"3. That it appears to render some exanthemata, *e. g.*, measles, milder than they would have been otherwise.

"4. That neither vaccination, inoculation, nor small-pox, guarantees the individual, in every instance from small-pox.

"5. That small-pox attacks some persons three times, or oftener.

"6. That there exist certain individuals who have perfect immunity from vaccination, inoculation, and small-pox.

"7. That great susceptibility to, or perfect immunity from, small-pox, is sometimes found to be common to several members of the same family."

